# Tribological Performance of a Paraffinic Base Oil Additive with Coated and Uncoated SiO_2_ Nanoparticles

**DOI:** 10.3390/ma17091993

**Published:** 2024-04-25

**Authors:** José M. Liñeira del Río, María J. G. Guimarey, Vanesa Somoza, Fátima Mariño, María J. P. Comuñas

**Affiliations:** 1 Laboratory of Thermophysical and Tribological Properties, Nafomat Group, Department of Applied Physics, Faculty of Physics and Instituto de Materiais (iMATUS), Universidade de Santiago de Compostela, 15782 Santiago de Compostela, Spain; mariajesus.guimarey@usc.es (M.J.G.G.); vanesa.somoza@rai.usc.es (V.S.); mariajp.comunas@usc.es (M.J.P.C.); 2 School of Engineering, University of the Basque Country UPV/EHU, Plaza Ingeniero Torres Quevedo 1, 48013 Bilbao, Spain; fatima.marino@ehu.eus

**Keywords:** friction, wear, nanolubricants, nanoparticle surface modification

## Abstract

Electric vehicles (EVs) have emerged as a technology that can replace internal combustion vehicles and reduce greenhouse gas emissions. Therefore, it is necessary to develop novel low-viscosity lubricants that can serve as potential transmission fluids for electric vehicles. Thus, this work analyzes the influence of both SiO_2_ and SiO_2_-SA (coated with stearic acid) nanomaterials on the tribological behavior of a paraffinic base oil with an ISO VG viscosity grade of 32 and a 133 viscosity index. A traditional two-step process through ultrasonic agitation was utilized to formulate eight nanolubricants of paraffinic oil + SiO_2_ and paraffinic base oil + SiO_2_-SA with nanopowder mass concentrations ranging from 0.15 wt% to 0.60 wt%. Visual control was utilized to investigate the stability of the nanolubricants. An experimental study of different properties (viscosity, viscosity index, density, friction coefficient, and wear) was performed. Friction analyses were carried out in pure sliding contacts at 393.15 K, and a 3D optical profilometer was used to quantify the wear. The friction results showed that, for the SiO_2_-SA nanolubricants, the friction coefficients were much lower than those obtained with the neat paraffinic base oil. The optimal nanoparticle mass concentration was 0.60 wt% SiO_2_-SA, with which the friction coefficient decreased by around 43%. Regarding wear, the greatest decreases in width, depth, and area were also found with the addition of 0.60 wt% SiO_2_-SA; thus, reductions of 21, 22, and 54% were obtained, respectively, compared with the neat paraffinic base oil.

## 1. Introduction

Energy needs are constantly increasing; consequently, the natural environment is significantly impacted. This is the case with the transport sector, as it is responsible for a large part of carbon dioxide, CO_2_, gas emissions, and climate change due to fuel-powered machinery [1]. For this reason, automotive industries need to develop new technologies to produce highly efficient vehicles for individual and public mobility [2,3]. Thus, electrification designs (hybrid or electric vehicles (EVs)) have emerged as an optimal solution for new propulsion systems to reduce greenhouse gas emissions [4], although a specific analysis of each country is necessary to ensure proper emissions reduction [5]. These greenhouse gas reductions, particularly that of CO_2_, greatly depend on the source of the electricity [6]. When electricity originates from renewable energy sources, the CO_2_ emissions of an EV are 4.5 times less than those of a combustion engine car [6]. Although EVs are very efficient and produce very low exhaust emissions, they have efficiency and endurance issues that affect the moving components and thus their tribology. Thus, tribological solutions such as new materials or optimized lubricants can help to increase the driving range of EVs since tribology can help to enhance the efficiency by lowering friction in elements like gears and wheel bearings [7].

Even though EVs exhibit significantly elevated efficiency in terms of energy use, there are still challenges related to the need to further enhance the efficiency; hence, the improvement of new fluids and materials [7] and the progression of new batteries [8] are being promoted. EVs require transmission oil lubricants with greater technical requirements [9] than those of internal combustion motors; this is because during the operation process contact is made with copper wires, sensors, and circuits [10]. Moreover, the high rotation rates of the electric motor require the use of lubricants with very low viscosity. If the oil viscosity is reduced, viscous drag and viscous heating drop and, therefore, the heat transfer is raised [9,11]. However, if the viscosity of a lubricant is lowered a shift from full film to boundary lubrication occurs and a more critical surface contact and wear is produced. This circumstance indicates that enhanced anti-wear and anti-friction properties are required. Therefore, to meet the needs of future EV lubricants, it is fundamental to use advanced additives [12]. The best conventional lubricants used for ICEVs, with the chance to be used in EV automotive elements, are made of mineral-based oils prepared with several additives to meet the rigorous requirements [7].

Recently, nanotechnology-based anti-friction and anti-wear additives were suggested for transmission fluids for EVs [7]. Then, the research on nanoparticles as oil additives was able to support the advance of a novel production of lubricants with low viscosity that were specifically modified to meet the necessities of EVs (electrified transmissions), owing to their outstanding anti-friction and anti-wear capacities, which can lead to an extended life in the operating conditions of EVs [12,13,14]. Furthermore, nanomaterials are more ecologically friendly than other conventional additives [15,16]. A crucial part of achieving a proper nanolubricant is the creation of temporal stability for an extended time; the sedimentation of nano-additives can lead to a decrease in efficiency and system damage owing to the abrasive wear [17]. To improve the stability of nanolubricants, different procedures can be carried out, using surfactants, physical treatment, or chemical surface modification [18,19]. The requirement of stable nanolubricants is particularly important for those lubricants composed of oils with low viscosity owing to the poor stability of the nanoparticles in such fluids. Even though lubricants with additives containing nanomaterials have exhibited good anti-friction and anti-wear performances in conventional lubricants [13,20,21,22], there is scarcely any investigation on nanolubricants with regard to EVs’ tribological needs. Mustafa et al. [9] have recently reviewed the tribological performance of several low-viscosity lubricants, based on different polyalphaolefin low-viscosity base oils and water. For instance, Chou et al. [23] analyzed the effect of adding Ni nanoparticles (20 nm) on the tribological activity of PAO6 base oil; they observed a reduction in friction between 7% and 30% and in wear between 5% and 45%, and they achieved the highest friction and wear reductions with the PAO6 + 0.5 wt% Ni nanolubricant. Because of the needs, it is necessary to improve and study potential stable lubricants formed through base oils with low viscosity and nano-additives. In this investigation, a paraffinic G-III base oil was selected to meet those qualities. The nano-additives used in this work, SiO_2_ nanoparticles, have exclusive physical and chemical characteristics; therefore, they can be used in several fields, such as in adhesives, the textile industry, and lubrication [24]. In fact, SiO_2_ as a lubricant additive usually shows an excellent anti-wear property, due to the fact that SiO_2_ has hydroxyl and unsaturated bonds and can form a solid chemical adsorption film to protect the metallic surface, significantly improving the friction performance of the lubricating oil. Furthermore, it has good electrical, optical, and magnetic properties and has received considerable interest in terms of applications such as those of catalysis, pharmaceuticals, drug delivery, and pigments. SiO_2_ nanopowder is a solid and colorless crystalline substance, which does not react with water and is resistant to acids. Furthermore, in this research, commercial SiO_2_ nanoparticles were chemically functionalized with stearic acid (SA) through an esterification process to enhance their stability in G-III base oil. SiO_2_ nanoparticles were studied as lubricant additives and demonstrated good ability as friction and wear modifiers [25,26,27,28,29,30]. For instance, Cortés et al. [25] studied the tribological performance of non-coated SiO_2_ nanoparticles, such as the additives of a vegetable oil, achieving decreases of up to 77% in terms of friction and 74% in terms of wear volume. Additionally, some authors [17,26] functionalized the SiO_2_ nanoparticle surface with the aim of enhancing the temporal stability of the nano-dispersions. For instance, Peng et al. [17] coated the SiO_2_ nanoparticles with oleic acid (SiO_2_-OA), reaching a temporal stability of about one month for a paraffin oil, with the mass percentages shifting from 0.05 to 1.0 wt% of the SiO_2_-OA.

In this article, we focus our attention on the use of commercial SiO_2_ and stearic acid-coated SiO_2_ (SiO_2_-SA) nanoparticles as additives of a G-III base oil, and these nanolubricants were tribologically analyzed at high temperature (393.15 K) in pure sliding contacts.

## 2. Materials and Methods

### 2.1. Base Oil and Nanoparticles

**Figure 1:**
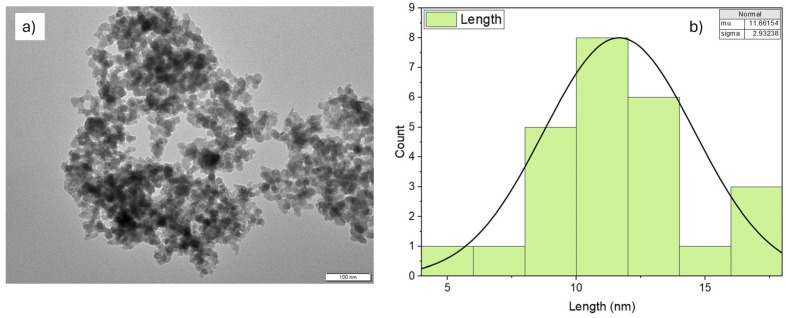
Image of TEM (**a**) and mean size particle distribution of SiO_2_ NPs (**b**).

The paraffinic G-III base oil was provided by Repsol S.A. (Madrid, Spain); it possesses a dynamic viscosity and density of 28.9 mPa and 0.8234 g·cm^−3^ at 313.15 K, respectively, and a 133 viscosity index. This oil was previously fully characterized through infrared spectroscopy (FTIR) and Raman spectroscopy; peaks associated with CH_3_ and CH_2_ stretching were observed using FTIR, and others attributed to C-H and C-C stretching were found using Raman spectroscopy [31]. Regarding the nano-additives, two different types of SiO_2_ nanoparticles were used. The first ones were commercial SiO_2_ nanoparticles provided by the company US Research Nanomaterials, Inc. (Houston, TX, USA), with a purity of 99% and a diameter of 8 nm. The second ones were the same SiO_2_ nanoparticles but coated in our laboratory with stearic acid (SiO_2_-SA). The SiO_2_ nanopowders were characterized by means of transmission electron microscopy (TEM); it can be seen in Figure 1a that the studied SiO_2_ NPs have a roughly spherical shape. Through the TEM characterization, the calculation of the average particle size was performed using Image J software (version 1.54h). Thus, as shown in Figure 1b, average sizes of around 11 nm were reached and were similar to the average size provided by the manufacturer (8 nm). Furthermore, in a previous work [32], infrared spectra of SA, uncoated SiO_2_ NPs, and SiO_2_-SA NPs were also reported; it was observed, among other information, that the characteristic peaks of SA also appear in the spectrum of SiO_2_-SA, evidencing a proper SA coating with the SiO_2_ nanoparticles.

### 2.2. Formulation of Nanolubricants

**Figure 2:**
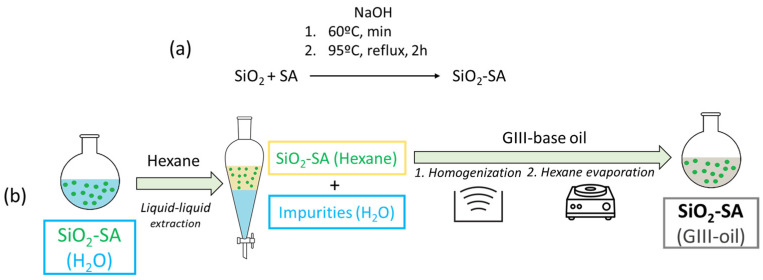
Scheme of the nanoparticle functionalization (**a**) and dispersion method (**b**).

The uncoated SiO_2_ nanolubricants were formulated with different mass concentrations of SiO_2_ (0.15, 0.30, 0.45 and 0.60 wt%) in G-III base oil. For this purpose, a conventional two-step method and a Sartorius MC 210P microbalance (±0.00001 g) were utilized. Furthermore, an ultrasonic method (Ultrasonic bath FB11203 Fisherbrand from Fisher Scientific, Hampton, VA, USA) was used for 4 h to homogenize the SiO_2_-based nanolubricants. On the other hand, to prepare the SiO_2_-SA nano-dispersions, commercial SiO_2_ nanopowders were coated with SA following the chemical reaction given in Figure 2a and then the dispersion method displayed in Figure 2b, to finally obtain a 4 wt% SiO_2_-SA nanolubricant. More details about a similar functionalization process can be seen in our previous article [32].

Therefore, dilutions of the achieved 4 wt% SiO_2_-SA nanolubricant were performed by adding G-III base oil, until reaching the desired (0.15, 0.30, 0.45, and 0.60 wt%) SiO_2_-SA nanolubricants. After the dilutions, the nanolubricants were also homogenized via an ultrasonic bath, as in the case of the bare SiO_2_ nanolubricants. Furthermore, the temporal stability of the nanolubricants was evaluated by visual control and refractive index evolution of the samples over time.

### 2.3. Thermophysical Characterization

The density of the nanolubricants was examined from 278.15 to 373.15 K, utilizing a vibrating densimeter Anton Paar (Graz, Austria) SVM 3000 Stabinger. The expanded (k = 2) uncertainty of the density measurements was 0.0005 g cm^−3^. The viscosity at atmospheric pressure and the viscosity index (VI) of the nanolubricants were also analyzed with the aforementioned densimeter. This device can measure kinematic and dynamic viscosities between 278.15 and 373.15 K. A relative expanded (k = 2) uncertainty of 1% was calculated for the dynamic viscosity.

### 2.4. Tribological Characterization

Friction tests were carried out in pure sliding contacts with a rheometer MCR 302 from Anton-Paar, kitted with a tribology unit T-PTD 200 and utilizing a Peltier hood H-PTD 200 for an ideal temperature control. In this research, a ball-on-three-pins test disposition was utilized; the ball is put on a shaft and set to turn by the rheometer motor, while being pushed at the same time against the three pins. The rheometer axial force is transferred into a normal force which proceeds perpendicularly to the contact positions on the pins. In this case, the ball turns on the pins below a 20 N normal force, resulting in a load of 9.43 N in each pin, which corresponds to a maximum contact pressure of around 0.8 GPa. Friction experiments were conducted at a constant rotational speed of 213 rpm and for 3400 s at 393.15 K. The specimens tested were polished AISI 52100 (100Cr6) steel balls (Ra = 20 nm) and pins (Ra = 50 nm) with a hardness of 62–66 HRC. The ball had a 12.7 mm diameter, and the cylindrical pins had a diameter and height that were both 6 mm. The balls and pins were cleaned with acetone/hexane and dried with air prior to the tribological tests. The pins were completely flooded by adding over 1.2 mL of each tested nanolubricant or base oil. At least three replicates were tested for each concentration of lubricant to obtain representative values. More information involving this tribological machine can be obtained from an earlier article [31]. To inspect the worn pins after the tribological studies, a 3D Optical Profiler was employed to measure the wear created in the pins for diverse parameters, such as wear scar diameter (WSD), wear track depth (WTD), or worn area. These parameters were analyzed in the three different pins by means of a confocal mode (10× objective). Moreover, a WITec alpha300R+ confocal Raman microscope (Oxford Instruments, Abingdon, UK) was utilized to obtain knowledge regarding the spreading of the nanoparticles in the worn pins.

## 3. Results

### 3.1. Stability of the Dispersions

**Figure 3:**
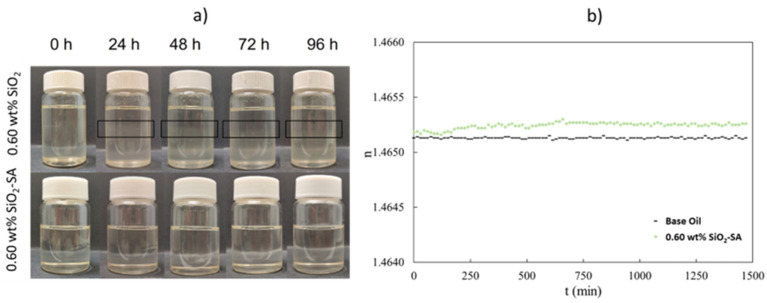
(**a**) Visual stability observation and (**b**) temporal evolution of the refractive index for SiO_2_-SA nanolubricant and base oil.

The stability of the SiO_2_ and SiO_2_-SA nanolubricants was checked using two different techniques: visual observation and temporal evolution of the refractive index using a Mettler Toledo RA-510 M refractometer (Columbus, OH, USA). Figure 3a reveals that sedimentation does not happen for the first 96 h after the nanolubricant formulation, for the coated SiO_2_-SA nanolubricants. Conversely, in the case of the uncoated SiO_2_ nanolubricants, it can be observed in Figure 3a that 24 h after the preparation, the sedimentation takes place. Thus, through the SA coating of SiO_2_ nanoparticles a better stability is reached. Similar stability behavior was observed in other studies using the stearic or oleic acid as the coating of the NPs, and stability improvements were achieved [19,33]. Figure 3b shows the temporal evolution of the refractive index (*n*) for the base oil and 0.6 wt% SiO_2_-SA nanolubricant. As can be seen, the tendencies of the refractive index evolution for the SiO_2_-SA nanolubricant and base oil are very similar, confirming a good stability against sedimentation.

### 3.2. Thermophysical Results

**Figure 4:**
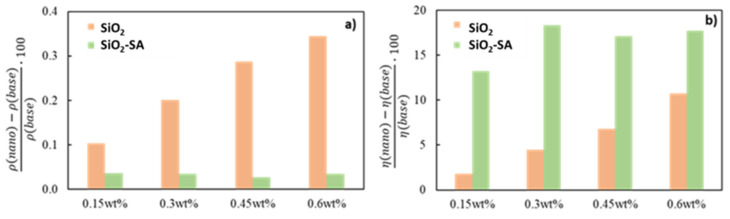
Relative increase in the densities (**a**) and viscosities (**b**) with the mass concentration with respect to the neat paraffinic base oil.

The experimental densities and dynamic viscosities acquired for the base oil and SiO_2_ and SiO_2_-SA nanolubricants are reported in Tables S1 and S2 (Supplementary Materials). Figure 4a shows the relative variation in the densities of the nanolubricant concentration with respect to the neat paraffinic base oil. For the SiO_2_ nanolubricants, a clear increase in density variation is observed as a function of the mass concentration of the nanoparticle; the higher the concentration, the higher the density of the nanolubricant. Thus, the SiO_2_ nanolubricants at 0.15, 0.3, 0.45 and 0.6 wt% increase relatively with respect to the neat base oil densities of 0.10, 0.20, 0.29 and 0.34%, respectively. The rise in nanolubricant density with the nanoparticle concentration is attributable to the agglomeration phenomenon [34]. However, for the SiO_2_-SA nanolubricants, the relative density increase is similar (around 0.03%) for all the concentrations of the functionalized nanoparticles. The relative viscosity variation in the SiO_2_ and SiO_2_-SA nanolubricants compared to the neat G-III paraffinic base oil is shown in Figure 4b. The dynamic viscosity rises as the concentration of SiO_2_ nanoparticles grows from 1% to 12%. Regarding the SiO_2_-SA nanoparticles, the growth in viscosity varies between 12 and 18% for the 0.15 and 0.3 wt% SiO_2_-SA nanolubricants, respectively.

**Figure 5:**
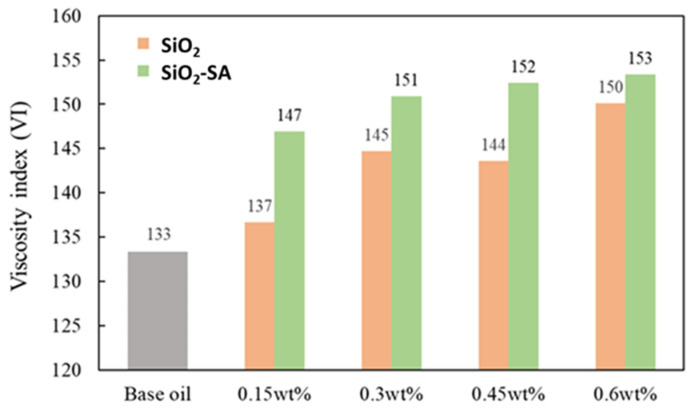
Viscosity index (VI) for the neat paraffinic base oil and for the SiO_2_ and SiO_2_-SA nanolubricants.

Additionally, with the aforementioned SiO_2_ and SiO_2_-SA nanolubricants, the impact of concentration on the viscosity index (VI) was analyzed, as shown in Figure 5. A suitable viscosity index (VI) is essential in a lubricant since it helps to avert collisions and friction among the mechanical device components during operation, while also enhancing the machine’s efficiency [35]. It can be observed that all the samples have a higher viscosity index (VI) than the neat base oil, which confirms that the nanolubricant remains useful even at elevated temperatures with the preservation of the thickness of the oil film. The results show that VI increased from 3% to 13% and from 11% to 15% for the SiO_2_ and SiO_2_-SA nanolubricants, respectively, compared with the neat base oil.

### 3.3. Tribological Results

**Figure 6:**
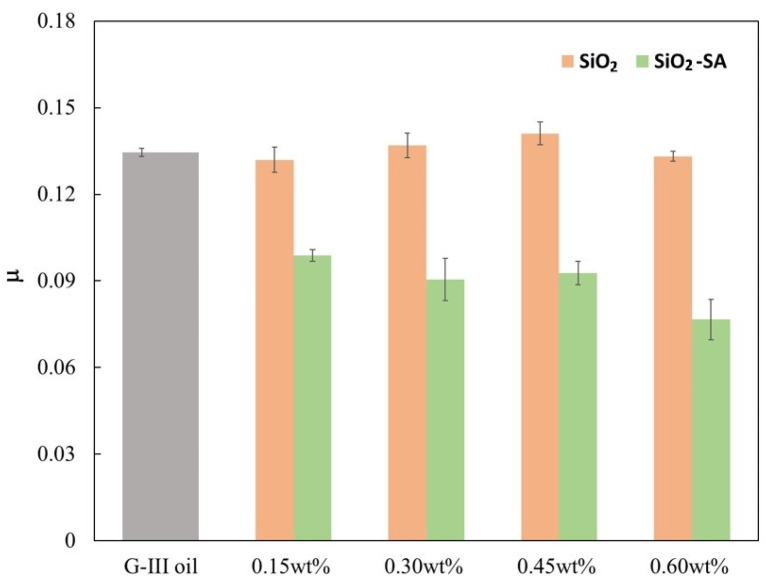
Mean friction coefficients, μ, for the prepared SiO_2_ and SiO_2_-SA nanolubricants and for the neat G-III base oil [31].

Figure 6 and Table 1 present the mean values of the coefficient of friction (*μ*) for all the tested lubricants based on G-III paraffinic base oil. The friction coefficients found for all the uncoated SiO_2_ nanolubricants are quite similar to that reached for the neat G-III base oil (without additives). Nonetheless, for the coated SiO_2_-SA nanolubricants the obtained friction coefficients are much lower than that previously reported using the neat G-III base oil [31]. Specifically, the optimal nanoparticle concentration was attained for the 0.60 wt% SiO_2_-SA nanolubricant, with a friction decrease of around 43% (*μ* of 0.077 was found against 0.134). This promising friction performance can be explained by the synergetic effect between the SiO_2_ nanoparticles and the coating of stearic acid.

As cited previously, the wear formed in the pins after the friction tests was estimated through many parameters of the wear track: width, depth, and area. For this goal, cross-section profiles and 3D mappings of the wear tracks were taken. The WSD, WTD, and transversal area mean values were taken from the profiles of the worn tracks on the pins tested with the nanolubricants and base oil. The values are reported in Table 1.

**Figure 7:**
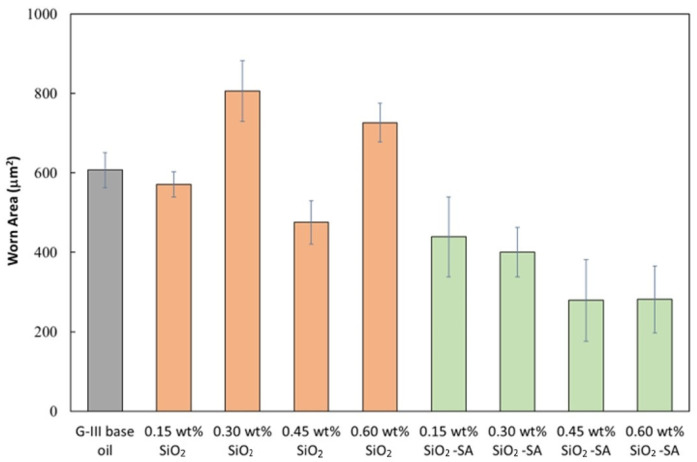
Mean worn areas, found for all the tested SiO_2_ and SiO_2_-SA nanolubricants and for the neat G-III base oil.

**Figure 8:**
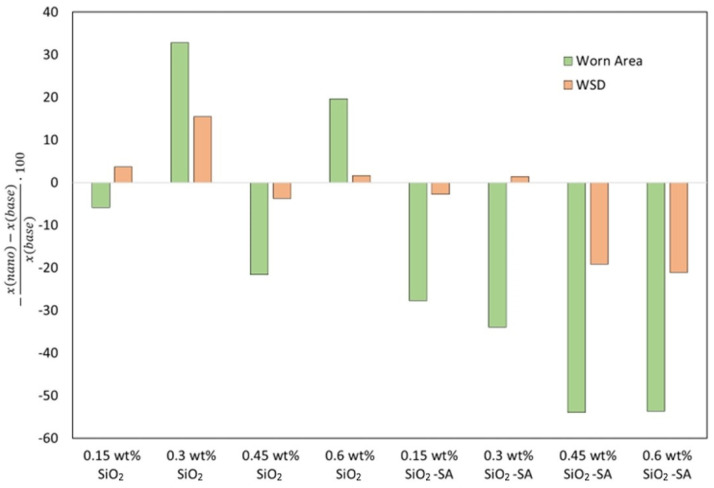
Mean reductions in WSD and worn area, obtained for all the tested SiO_2_ and SiO_2_-SA nanolubricants.

As with the friction results, the SiO_2_ nanolubricants revealed similar wear results to those of the G-III base oil. Nonetheless, for all the SiO_2_-SA-based nanolubricants, the produced wear was greatly inferior to that achieved with neat G-III base oil, particularly in the case of the worn areas (Figure 7). Furthermore, the additive mass concentration used in the nanolubricant design considerably influenced the lubrication performance. Specifically, the greatest decreases in width and area were reached with the G-III base oil + 0.60 wt% SiO_2_-SA nanolubricant (Table 1), with reductions of 21 and 54%, respectively (Figure 8).

Similar improved tribological performances with SiO_2_ NPs were previously obtained by other authors. Thus, Sanukrishna et al. [29] studied the tribological properties of SiO_2_ NPs as additives of a PAG lubricant, observing friction reductions of around 38% and wear reductions of 41%. Also, Rastogi et al. [30] studied the effect of SiO_2_ nanoparticles on the tribological characteristics of jatropha oil, obtaining important friction and wear reductions for different normal loads.

**Figure 9:**
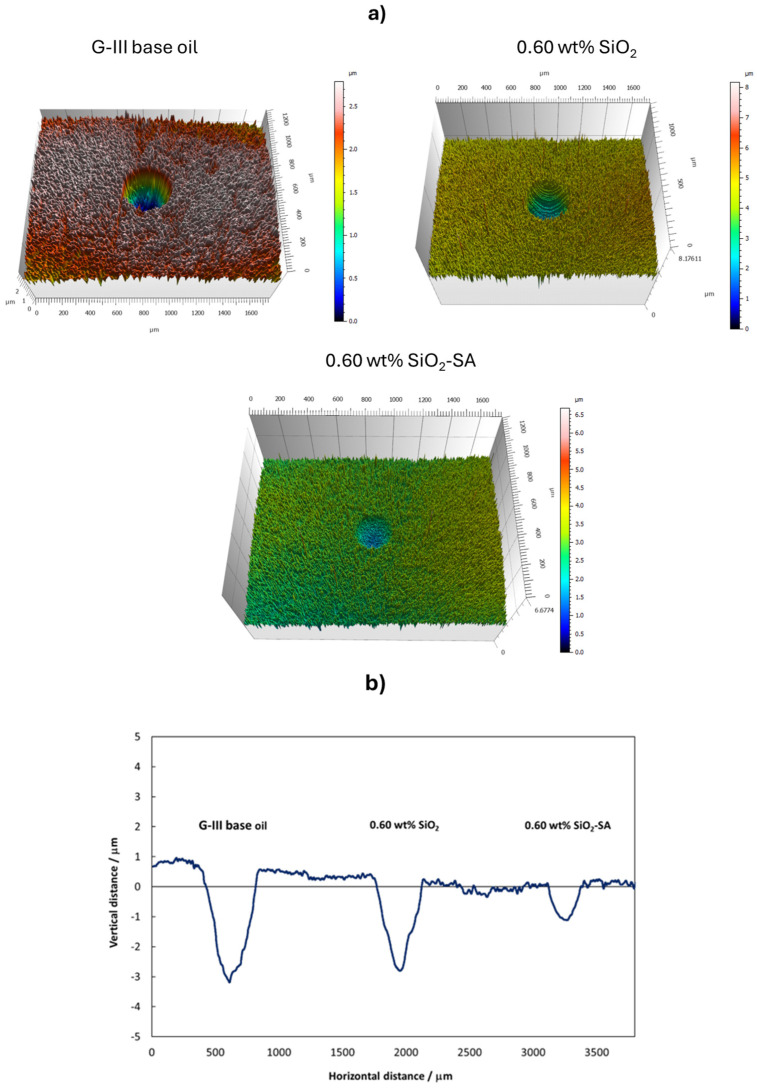
Three-dimensional (**a**) and two-dimensional (**b**) profiles of worn pins tested with neat G-III base oil [31], 0.60 wt% SiO_2_, and 0.60 wt% SiO_2_-SA.

Additionally, it can be clearly observed in the worn profiles in Figure 9 that the optimal SiO_2_ nanolubricant that contains the stearic acid coating presents considerably better anti-wear capacities with respect to the G-III base oil and the optimal uncoated SiO_2_ nanolubricant.

**Figure 10:**
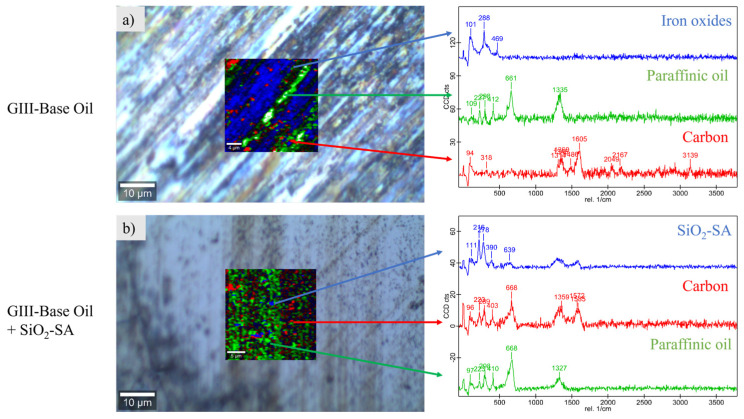
Mapping of Raman with worn pins tested with G-III base oil and 0.60 wt% SiO_2_-SA.

Furthermore, the Raman spectra of the nanolubricant components evidence the fact that characteristic areas of these elements appear in the worn surfaces of the pins (Figure 10). Thus, in Figure 10a blue areas of Raman mapping are associated with iron oxides, green areas with the base oil, and red areas with the burned oil. In Figure 10b, it can be seen from the presence of blue areas associated with the SiO_2_-SA nanoparticles that the spectrum of this area coincides with the SiO_2_-SA Raman spectrum [32]. Considering these Raman analyses, a protective tribofilm from the SiO_2_-SA in the tribo-contact can be a possible tribological mechanism that participates in the decrease in friction and wear. Thus, the spherical SiO_2_ NPs that are dispersed in the paraffinic lubricant with the high contact pressure can enter into the interspace of contact surfaces and progressively deposit on surfaces, causing the creation of a physical film. This tribofilm can separate the two metal surfaces and prevent direct contact [36]. Furthermore, some tribochemical reactions can occur, boosted by the high temperatures and pressures caused by the friction process. Hence, these conditions could cause the breaking of the bonds between SA and the coated SiO_2_ NPs, as was previously pointed out by Zhang et al. [37] for SA-modified TiO_2_ NPs. SiO_2_ NPs can easily be adsorbed on the worn surface, generating a boundary-lubricating film, whereas the SA can also be physically adsorbed on the steel surface during the tribotests, generating good lubricant properties [38,39]. Likewise, owing to the spherical nature of SiO_2_ NPs, they are more likely to roll between two surfaces, reducing the friction coefficient and wear. Therefore, rolling and tribofilm formation are the two possible tribological mechanisms. Similar results were previously obtained by Xie et al. [40] for SiO_2_ NPs dispersed in engine oil.

## 4. Conclusions

The formulation and tribological characterization of modified SiO_2_ nanolubricants was performed with the aim of contributing to the EV industry demands for developed lubricants that can be used in the future as EV transmission fluids, leading to an improvement in their energy efficiency. Therefore, the main conclusions of this investigation are:

- Good stability against sedimentation was achieved with SiO_2_ nanoparticles, coated with stearic acid and dispersed in a paraffinic base oil.
- Friction coefficients found with the SiO_2_-SA nanolubricants are reduced in comparison with the neat G-III base oil.
- The wear produced in the pins is lower than that found with G-III base oil; the optimal nanolubricant is the G-III base oil + 0.60 wt% SiO_2_-SA, with reductions of up to 21, 22, and 54% in terms of width, depth, and area, respectively.
- The tribological lubrication mechanisms can be justified by the rolling and tribofilm formation of the SiO_2_-SA nanoparticles.
- Before applying these nanolubricants in EVs, more studies are needed, such as tribological tests for extended times to estimate their possible degradation and the study of other key properties, such as thermal or electrical conductivity, and the compatibility and synergies with other additives used in the lubricant formulation.

## Figures and Tables

**Table 1:** Average coefficients of friction, *μ*, and mean parameters of wear with their standard deviations for the studied G-III base oil nanolubricants at 393.15 K.

Sample	μ	σ	WSD/μm	σ/μm	WTD/μm	σ/μm	Area/μm^2^	σ/μm^2^
G-III base oil [31]	0.1351	0.0014	366	18	2.11	0.19	607	44
+0.15 wt% SiO_2_	0.1319	0.0011	380	23	2.76	0.72	571	31
+0.30 wt% SiO_2_	0.1370	0.0010	422	53	3.37	0.67	806	76
+0.45 wt% SiO_2_	0.1410	0.0011	352	31	2.61	0.45	476	55
+0.60 wt% SiO_2_	0.1332	0.0012	372	8.3	2.67	0.46	726	49
+0.15 wt% SiO_2_-SA	0.0989	0.0014	356	23	2.00	0.49	439	99
+0.30 wt% SiO_2_-SA	0.0905	0.0011	371	18	1.60	0.19	401	62
+0.45 wt% SiO_2_-SA	0.0927	0.0010	296	54	1.80	0.38	279	98
+0.60 wt% SiO_2_-SA	0.0766	0.0011	289	25	1.64	0.47	281	84

## Data Availability

Data are contained within the article and Supplementary Materials.

## Supplementary Materials

The following supporting information can be downloaded at: https://www.mdpi.com/article/10.3390/ma17091993/s1, Table S1: Experimental density determined with Stabinger densimeter for the paraffinic base oil and the nanolubricants at different temperatures and at 0.0991 MPa, Table S2: Experimental viscosity determined with Stabinger rotational viscometer for the paraffinic base oil and the nanolubricants at 0.0991 MPa at different temperatures.

## References

[1] Liu Z., Ciais P., Deng Z., Davis S.J., Zheng B., Wang Y., Cui D., Zhu B., Dou X., Ke P. (2020). Carbon Monitor, a near-real-time daily dataset of global CO_2_ emission from fossil fuel and cement production. Sci. Data.

[2] Alanazi F. (2023). Electric Vehicles: Benefits, Challenges, and Potential Solutions for Widespread Adaptation. Appl. Sci..

[3] Cao J., Chen X., Qiu R., Hou S. (2021). Electric vehicle industry sustainable development with a stakeholder engagement system. Technol. Soc..

[4] Xu L., Yilmaz H.Ü., Wang Z., Poganietz W.-R., Jochem P. (2020). Greenhouse gas emissions of electric vehicles in Europe considering different charging strategies. Transp. Res. Part D Transp. Environ..

[5] Fusco Rovai F., Regina da Cal Seixas S., Keutenedjian Mady C.E. (2023). Regional energy policies for electrifying car fleets. Energy.

[6] Holmberg K., Erdemir A. (2019). The impact of tribology on energy use and CO_2_ emission globally and in combustion engine and electric cars. Tribol. Int..

[7] Farfan-Cabrera L.I. (2019). Tribology of electric vehicles: A review of critical components, current state and future improvement trends. Tribol. Int..

[8] Martí-Florences M., Cecilia A., Costa-Castelló R. (2023). Modelling and Estimation in Lithium-Ion Batteries: A Literature Review. Energies.

[9] Ahmed Abdalglil Mustafa W., Dassenoy F., Sarno M., Senatore A. (2022). A review on potentials and challenges of nanolubricants as promising lubricants for electric vehicles. Lubr. Sci..

[10] Parenago O.P., Lyadov A.S., Maksimov A.L. (2022). Development of Lubricant Formulations for Modern Electric Vehicles. Russ. J. Appl. Chem..

[11] Narita K., Takekawa D. (2019). Lubricants Technology Applied to Transmissions in Hybrid Electric Vehicles and Electric Vehicles.

[12] Tung S.C., Woydt M., Shah R. (2020). Global Insights on Future Trends of Hybrid/EV Driveline Lubrication and Thermal Management. Front. Mech. Eng..

[13] Chen Y., Jha S., Raut A., Zhang W., Liang H. (2020). Performance Characteristics of Lubricants in Electric and Hybrid Vehicles: A Review of Current and Future Needs. Front. Mech. Eng..

[14] Kim H.-J., Seo K.-J., Kang K.H., Kim D.-E. (2016). Nano-lubrication: A review. Int. J. Precis. Eng. Manuf..

[15] Dai W., Kheireddin B., Gao H., Liang H. (2016). Roles of nanoparticles in oil lubrication. Tribol. Int..

[16] Berman D., Erdemir A., Sumant A.V. (2014). Graphene: A new emerging lubricant. Mater. Today.

[17] Peng D.X., Kang Y., Hwang R.M., Shyr S.S., Chang Y.P. (2009). Tribological properties of diamond and SiO_2_ nanoparticles added in paraffin. Tribol. Int..

[18] Azman N.F., Samion S. (2019). Dispersion Stability and Lubrication Mechanism of Nanolubricants: A Review. Int. J. Precis. Eng. Manuf.-Green Technol..

[19] Mariño F., Liñeira del Río J.M., López E.R., Fernández J. (2023). Chemically modified nanomaterials as lubricant additive: Time stability, friction, and wear. J. Mol. Liq..

[20] Liñeira del Río J.M., López E.R., Fernández J., García F. (2019). Tribological properties of dispersions based on reduced graphene oxide sheets and trimethylolpropane trioleate or PAO 40 oils. J. Mol. Liq..

[21] Wang B., Qiu F., Barber G.C., Zou Q., Wang J., Guo S., Yuan Y., Jiang Q. (2022). Role of nano-sized materials as lubricant additives in friction and wear reduction: A review. Wear.

[22] Singh A., Chauhan P., Mamatha T.G. (2020). A review on tribological performance of lubricants with nanoparticles additives. Mater. Today Proc..

[23] Chou R., Battez A.H., Cabello J.J., Viesca J.L., Osorio A., Sagastume A. (2010). Tribological behavior of polyalphaolefin with the addition of nickel nanoparticles. Tribol. Int..

[24] Dubey R.S., Rajesh Y.B.R.D., More M.A. (2015). Synthesis and Characterization of SiO_2_ Nanoparticles via Sol-gel Method for Industrial Applications. Mater. Today Proc..

[25] Cortes V., Sanchez, Gonzalez R., Alcoutlabi M., Ortega J. (2020). The Performance of SiO_2_ and TiO_2_ Nanoparticles as Lubricant Additives in Sunflower Oil. Lubricants.

[26] Sui T., Song B., Wen Y.-h., Zhang F. (2016). Bifunctional hairy silica nanoparticles as high-performance additives for lubricant. Sci. Rep..

[27] Jiao D., Zheng S., Wang Y., Guan R., Cao B. (2011). The tribology properties of alumina/silica composite nanoparticles as lubricant additives. Appl. Surf. Sci..

[28] Peng D.X., Chen C.H., Kang Y., Chang Y.P., Chang S.Y. (2010). Size effects of SiO nanoparticles as oil additives on tribology of lubricant. Ind. Lubr. Tribol..

[29] Sanukrishna S.S., Shafi M., Murukan M., Jose Prakash M. (2019). Effect of SiO_2_ nanoparticles on the heat transfer characteristics of refrigerant and tribological behaviour of lubricant. Powder Technol..

[30] Mohan Rastogi P., Kumar R., Kumar N. (2021). Effect of SiO_2_ nanoparticles on the tribological characteristics of jatropha oil. Mater. Today Proc..

[31] Liñeira del Río J.M., Rial R., López E.R., Fernández J. (2022). Tribological enhancement using Mn_3_O_4_-Graphene nanocomposites as additives for potential transmission fluids of electric vehicles. J. Mol. Liq..

[32] Mariño F., Liñeira del Río J.M., Gonçalves D.E.P., Seabra J.H.O., López E.R., Fernández J. (2023). Effect of the addition of coated SiO_2_ nanoparticles on the tribological behavior of a low-viscosity polyalphaolefin base oil. Wear.

[33] Liñeira del Río J.M., Mariño F., López E.R., Gonçalves D.E.P., Seabra J.H.O., Fernández J. (2023). Tribological enhancement of potential electric vehicle lubricants using coated TiO_2_ nanoparticles as additives. J. Mol. Liq..

[34] Kannaiyan S., Boobalan C., Umasankaran A., Ravirajan A., Sathyan S., Thomas T. (2017). Comparison of experimental and calculated thermophysical properties of alumina/cupric oxide hybrid nanofluids. J. Mol. Liq..

[35] Ali M.K.A., Xianjun H., Mai L., Qingping C., Turkson R.F., Bicheng C. (2016). Improving the tribological characteristics of piston ring assembly in automotive engines using Al_2_O_3_ and TiO_2_ nanomaterials as nano-lubricant additives. Tribol. Int..

[36] Kato H., Komai K. (2007). Tribofilm formation and mild wear by tribo-sintering of nanometer-sized oxide particles on rubbing steel surfaces. Wear.

[37] Zhang L., Chen L., Wan H., Chen J., Zhou H. (2011). Synthesis and Tribological Properties of Stearic Acid-Modified Anatase (TiO_2_) Nanoparticles. Tribol. Lett..

[38] Zachariah Z., Nalam P.C., Ravindra A., Raju A., Mohanlal A., Wang K., Castillo R.V., Espinosa-Marzal R.M. (2019). Correlation Between the Adsorption and the Nanotribological Performance of Fatty Acid-Based Organic Friction Modifiers on Stainless Steel. Tribol. Lett..

[39] Sahoo R.R., Biswas S.K. (2009). Frictional response of fatty acids on steel. J. Colloid Interface Sci..

[40] Xie H., Jiang B., He J., Xia X., Pan F. (2016). Lubrication performance of MoS_2_ and SiO_2_ nanoparticles as lubricant additives in magnesium alloy-steel contacts. Tribol. Int..

