# Supplementary material for: Tribological Performance of a Paraffinic Base Oil Additive with Coated and Uncoated SiO2 Nanoparticles
Source: Materials (Basel). 2024 Apr 25;17(9):1993. doi: 10.3390/ma17091993 (PMC11084597; doi:10.3390/ma17091993)
Supplement: Supplementary file 1 [file materials-17-01993-s001.zip › materials-2941863-supplementary.pdf]

materials

MDPI

Supplementary material

Tribological Performance of a Paraffinic Base Oil Additivated with Coated and Uncoated SiO_2 Nanoparticles

José M. Liñeira del Río [1,*], María J. G. Guimarey [1], Vanesa Somoza [1], Fátima Mariño [2] and María J. P. Comuñas [1]

1 Laboratory of Thermophysical and Tribological Properties, Nafomat Group, Department of Applied Physics, Faculty of Physics and Instituto de Materiais (iMATUS), Universidade de Santiago de Compostela, 15782 Santiago de Compostela, Spain; mariajesus.guimarey@usc.es (M.J.G.G.); vanesa.somoza@rai.usc.es (V.S.); mariajp.comunas@usc.es (M.J.P.C.)

2 School of Engineering, University of the Basque Country UPV/EHU, Plaza Ingeniero Torres Quevedo 1, 48013 Bilbao, Spain; fatima.marino@ehu.eus

* Correspondence: josemanuel.lineira@usc.es

Table S1: Experimental density, ρ^a, determined with Stabinger densimeter for the paraffinic base oil and the nanolubricants at different temperatures, T^b, and 0.0991 MPa^c.

T/K	ρ/g·cm^{-3}	T/K	ρ/g·cm^{-3}	T/K	ρ/g·cm^{-3}
		Paraffinic base oil			
278.15	0.8447	313.15	0.8234	348.15	0.8020
283.15	0.8417	318.15	0.8204	353.15	0.7989
288.15	0.8387	323.15	0.8173	358.15	0.7958
293.15	0.8356	328.15	0.8143	363.15	0.7927
298.15	0.8326	333.15	0.8112	368.15	0.7896
303.15	0.8295	338.15	0.8081	373.15	0.7865
308.15	0.8265	343.15	0.8050		
		Paraffinic base oil + 0.15 wt% SiO_2			
278.15	0.8458	313.15	0.8244	348.15	0.8027
283.15	0.8427	318.15	0.8213	353.15	0.7996
288.15	0.8397	323.15	0.8182	358.15	0.7965
293.15	0.8366	328.15	0.8151	363.15	0.7933
298.15	0.8336	333.15	0.8120	368.15	0.7902
303.15	0.8305	338.15	0.8089	373.15	0.7871
308.15	0.8275	343.15	0.8058		
		Paraffinic base oil + 0.30 wt% SiO_2			
278.15	0.8466	313.15	0.8252	348.15	0.8035
283.15	0.8435	318.15	0.8221	353.15	0.8004
288.15	0.8405	323.15	0.8190	358.15	0.7972
293.15	0.8374	328.15	0.8159	363.15	0.7941
298.15	0.8344	333.15	0.8128	368.15	0.7910
303.15	0.8313	338.15	0.8097	373.15	0.7879

308.15	0.8283	343.15	0.8066		
Paraffinic base oil + 0.45 wt% SiO_2					
278.15	0.8473	313.15	0.8259	348.15	0.8042
283.15	0.8442	318.15	0.8228	353.15	0.8011
288.15	0.8412	323.15	0.8197	358.15	0.7979
293.15	0.8381	328.15	0.8166	363.15	0.7948
298.15	0.8351	333.15	0.8135	368.15	0.7917
303.15	0.8320	338.15	0.8104	373.15	0.7886
308.15	0.8290	343.15	0.8073		
Paraffinic base oil + 0.60 wt% SiO_2					
278.15	0.8476	313.15	0.8263	348.15	0.8047
283.15	0.8446	318.15	0.8232	353.15	0.8016
288.15	0.8415	323.15	0.8202	358.15	0.7985
293.15	0.8385	328.15	0.8171	363.15	0.7954
298.15	0.8355	333.15	0.8140	368.15	0.7923
303.15	0.8324	338.15	0.8109	373.15	0.7892
308.15	0.8294	343.15	0.8078		
Paraffinic base oil + 0.15 wt% SiO_2 − SA					
278.15	0.8451	313.15	0.8238	348.15	0.8022
283.15	0.8421	318.15	0.8207	353.15	0.7991
288.15	0.8390	323.15	0.8177	358.15	0.7960
293.15	0.8360	328.15	0.8146	363.15	0.7929
298.15	0.8329	333.15	0.8115	368.15	0.7898
303.15	0.8299	338.15	0.8084	373.15	0.7867
308.15	0.8268	343.15	0.8053		
Paraffinic base oil + 0.30 wt% SiO_2 − SA					
278.15	0.8452	313.15	0.8238	348.15	0.8021
283.15	0.8421	318.15	0.8207	353.15	0.7990
288.15	0.8391	323.15	0.8177	358.15	0.7959
293.15	0.8360	328.15	0.8146	363.15	0.7928
298.15	0.8330	333.15	0.8115	368.15	0.7897
303.15	0.8300	338.15	0.8084	373.15	0.7866
308.15	0.8269	343.15	0.8052		
Paraffinic base oil + 0.45 wt% SiO_2 − SA					
278.15	0.8452	313.15	0.8238	348.15	0.8020
283.15	0.8421	318.15	0.8207	353.15	0.7989
288.15	0.8391	323.15	0.8176	358.15	0.7958
293.15	0.8360	328.15	0.8145	363.15	0.7927
298.15	0.8330	333.15	0.8114	368.15	0.7896
303.15	0.8299	338.15	0.8083	373.15	0.7865
308.15	0.8269	343.15	0.8051		

		Paraffinic base oil + 0.60 wt% SiO_2 – SA			
278.15	0.8451	313.15	0.8238	348.15	0.8022
283.15	0.8420	318.15	0.8207	353.15	0.7991
288.15	0.8390	323.15	0.8177	358.15	0.7960
293.15	0.8360	328.15	0.8146	363.15	0.7929
298.15	0.8329	333.15	0.8115	368.15	0.7897
303.15	0.8299	338.15	0.8084	373.15	0.7866
308.15	0.8268	343.15	0.8053		

[a] Combined expanded density uncertainty is $U_c(\rho) = 5\cdot10^{-4}$ g·cm^{-3}; [b] expanded temperature uncertainty is $U(T) = 0.02$ K and [c] expanded pressure uncertainty is $U(p) = 0.0005$ MPa (0.95 level of confidence).

Table S2 Experimental viscosity, η^a, determined with Stabinger rotational viscometer for the paraffinic base oil and the nanolubricants at 0.0991 MPa[b] at different temperatures T^c.

T/K	η/mPa·s	T/K	η/mPa·s	T/K	η/mPa·s
		Paraffinic base oil			
278.15	180.78	313.15	28.850	348.15	8.9519
283.15	131.55	318.15	23.626	353.15	7.8537
288.15	97.820	323.15	19.589	358.15	6.9407
293.15	74.123	328.15	16.432	363.15	6.1776
298.15	57.367	333.15	13.924	368.15	5.5339
303.15	44.838	338.15	11.910	373.15	4.9905
308.15	35.706	343.15	10.285		
		Paraffinic base oil + 0.15 wt% SiO_2			
278.15	184.09	313.15	29.334	348.15	9.1231
283.15	133.95	318.15	24.021	353.15	8.0063
288.15	99.599	323.15	19.915	358.15	7.0747
293.15	75.439	328.15	16.702	363.15	6.2992
298.15	58.350	333.15	14.174	368.15	5.6439
303.15	45.600	338.15	12.132	373.15	5.0860
308.15	36.310	343.15	10.476		
		Paraffinic base oil + 0.30 wt% SiO_2			
278.15	189.99	313.15	30.111	348.15	9.3296
283.15	138.16	318.15	24.649	353.15	8.1934
288.15	102.63	323.15	20.420	358.15	7.2505
293.15	77.663	328.15	17.108	363.15	6.4561
298.15	60.011	333.15	14.496	368.15	5.7882
303.15	46.879	338.15	12.407	373.15	5.2189
308.15	37.304	343.15	10.708		
		Paraffinic base oil + 0.45 wt% SiO_2			
278.15	195.07	313.15	30.819	348.15	9.4995
283.15	142.06	318.15	25.196	353.15	8.3351
288.15	105.50	323.15	20.847	358.15	7.3777

293.15	79.808	328.15	17.453	363.15	6.5733
298.15	61.634	333.15	14.787	368.15	5.8965
303.15	48.100	338.15	12.637	373.15	5.3191
308.15	38.222	343.15	10.904		
Paraffinic base oil + 0.60 wt% SiO_2					
278.15	203.22	313.15	31.866	348.15	9.8839
283.15	146.84	318.15	26.035	353.15	8.6905
288.15	108.97	323.15	21.540	358.15	7.6958
293.15	82.378	328.15	18.058	363.15	6.8634
298.15	63.609	333.15	15.290	368.15	6.1547
303.15	49.668	338.15	13.108	373.15	5.5460
308.15	39.506	343.15	11.341		
Paraffinic base oil + 0.15 wt% $SiO_2 - SA$					
278.15	203.60	313.15	32.636	348.15	10.171
283.15	148.17	318.15	26.736	353.15	8.9304
288.15	110.22	323.15	22.179	358.15	7.9086
293.15	83.576	328.15	18.604	363.15	7.0083
298.15	64.656	333.15	15.773	368.15	6.3092
303.15	50.582	338.15	13.513	373.15	5.6613
308.15	40.386	343.15	11.677		
Paraffinic base oil + 0.30 wt% $SiO_2 - SA$					
278.15	213.19	313.15	34.157	348.15	10.585
283.15	155.15	318.15	27.979	353.15	9.2876
288.15	115.53	323.15	23.175	358.15	8.2184
293.15	87.628	328.15	19.393	363.15	7.3156
298.15	67.820	333.15	16.444	368.15	6.6231
303.15	53.108	338.15	14.073	373.15	5.9662
308.15	42.279	343.15	12.154		
Paraffinic base oil + 0.45 wt% $SiO_2 - SA$					
278.15	211.33	313.15	33.688	348.15	10.508
283.15	153.90	318.15	27.593	353.15	9.2334
288.15	114.21	323.15	22.859	358.15	8.1706
293.15	86.519	328.15	19.210	363.15	7.2776
298.15	66.875	333.15	16.291	368.15	6.5231
303.15	52.301	338.15	13.953	373.15	5.8877
308.15	41.671	343.15	12.057		
Paraffinic base oil + 0.60 wt% $SiO_2 - SA$					
278.15	211.90	313.15	33.949	348.15	10.570
283.15	154.21	318.15	27.809	353.15	9.2784
288.15	114.78	323.15	23.066	358.15	8.1988
293.15	86.980	328.15	19.350	363.15	7.2938
298.15	67.303	333.15	16.404	368.15	6.5358

303.15	52.697	338.15	14.040	373.15	5.9087
308.15	41.993	343.15	12.127		

[a] Combined relative expanded viscosity uncertainty is $U_c(\eta)$ = 1%; [b] expanded pressure uncertainty is $U(p)$ = 0.0005 MPa and [c] expanded temperature uncertainty is $U(T)$ = 0.02 K (0.95 level of confidence).
